# Functional characterization of *Candida* *albicans* Hos2 histone deacetylase

**DOI:** 10.12688/f1000research.2-238.v3

**Published:** 2014-07-22

**Authors:** G Karthikeyan, Maneesh Paul-Satyaseela, Nachiappan Dhatchana Moorthy, Radha Gopalaswamy, Shridhar Narayanan

**Affiliations:** 1 Drug Discovery Research, Orchid Chemicals and Pharmaceuticals Limited, Chennai, 600 119, India; 2 Current address: Samrud Foundation for Health & Research, Bangalore, 560106, India; 3 Current address: AstraZeneca India Pvt. Ltd, Bengaluru, 560024, India

## Abstract

*Candida albicans* is a mucosal commensal organism capable of causing superficial (oral and vaginal thrush) infections in immune normal hosts, but is a major pathogen causing systemic and mucosal infections in immunocompromised individuals. Azoles have been very effective anti-fungal agents and the mainstay in treating opportunistic mold and yeast infections. Azole resistant strains have emerged compromising the utility of this class of drugs. It has been shown that azole resistance can be reversed by the co-administration of a histone deacetylase (HDAC) inhibitor, suggesting that resistance is mediated by epigenetic mechanisms possibly involving Hos2, a fungal deacetylase. We report here the cloning and functional characterization of *HOS2 (H*igh *O*smolarity *S*ensitive) *,* a gene coding for fungal histone deacetylase from *C. albicans*. Inhibition studies showed that Hos2 is susceptible to pan inhibitors such as trichostatin A (TSA) and suberoylanilide hydroxamic acid (SAHA), but is not inhibited by class I inhibitors such as MS-275. This *in* *vitro* enzymatic assay, which is amenable to high throughput could be used for screening potent fungal Hos2 inhibitors that could be a potential anti-fungal adjuvant. Purified Hos2 protein consistently deacetylated tubulins, rather than histones from TSA-treated cells. Hos2 has been reported to be a putative NAD+ dependent histone deacetylase, a feature of sirtuins. We assayed for sirtuin activation with resveratrol and purified Hos2 protein and did not find any sirtuin activity.

## Introduction

*Candida albicans* is a commensal organism found in the mucosa and gastrointestinal tract of most healthy individuals but can cause superficial (oral and vaginal thrush) infections in immune-normal hosts and severe systemic infection in immunocompromised patients ^1^. This fungus is of clinical importance and is one of the leading causes of systemic infections in immunocompromised individuals. *C. albicans* is the fourth most common cause of nosocomial bloodstream infections and is associated with high mortality rates ^2^. Azoles and echinocandins targeting the ergosterol and cell wall biosynthesis pathway respectively have been used as anti-fungal drugs though the emergence of drug-resistant strains has compromised the efficacy and utility of these drugs ^3^.

Azole resistance in *Candida* sp. is mediated by up regulation of genes encoding *ERG11*, a lanosterol demethylase ^4– 6^, *MDR1* ^5, 7^ and by *CDR* (Candida drug resistance) efflux pumps ^5, 7– 9^. A combination of existing anti-fungals with new classes of drugs that act by different mechanisms will be viable alternatives to the current monotherapy regimen, which contributes to the emergence of drug resistance.

Histone deacetylases (HDAC) play an important role in modulating chromatin conformation, by deacetylating crucial lysine residues in the histone octamers over which the chromatin DNA are wrapped ^10^. Human HDAC`s fall into four broad categories, Class I (HDAC1, 2, 3, and 8), Class II a (HDAC 4, 5, 7 and 9) Class II b (HDAC 6 and 10) Class III (sirtuins) and Class IV (HDAC11) based on sequence homology, substrate preference and co-factor requirements. The involvement of each of these isoforms in disease pathology has been elucidated to some extent in recent times. The approval of suberoylanilide hydroxamic acid (SAHA) ^11^, a well known inhibitor of HDACs by the US FDA for treating CTCL, (cutaneous T cell lymphoma) ^12, 13^ has thrown open the doors for exploring the use of HDAC inhibitors in combination with existing drugs for several diseases, such as malaria and Kala-azar etc., ^14– 16^.

Class specific inhibitors are now becoming a reality for human HDAC isoforms. For example the HDAC Class I specific inhibitor MS-275 is in advanced clinical trials (clinical trial Nos. NCT00020579, NCT00866333) for several forms of cancer, and the HDAC Class II specific inhibitor ACY-1215 is at an advanced clinical phase (clinical trial Nos. NCT01323751, NCT01583283) for myeloma.

HDAC inhibitors have been shown to synergize the actions of antifungal agents, due to their effect on preventing drug resistance *in vitro* ^17, 18^. Therefore, an alternative approach to address fungal drug resistance could be to harness the potential of modulating fungal gene expression by inhibition of fungal HDACs ^17, 19^.

Hos2, a Class I HDAC enzyme plays an important role in gene activation in the yeast *Saccharomyces cerevisiae* by binding to open reading frames (ORFs) of active genes ^20^. Hos2/Set3 histone deacetylase complex (Set3C) plays a key role in the conversion of white phase to virulent opaque phase in *C. albicans* ^21^. Deletion of Hos2, the catalytic subunit of the Set3 complex produced a phenotype resembling inhibition of the Set3C by Trichostatin-A (TSA) ^22^. Serum-induced morphogenesis of some *C. albicans* strains was shown to be inhibited by TSA ^19^. Thus inhibiting the morphogenetic ability of this opportunistic pathogen using HDAC inhibitors holds the promise of future antifungal agents. Recently a small molecule, MGCD 290 (Hos2 inhibitor) has entered clinical trials (clinical trial number NCT01497223)) for use in combination with azoles, such as fluconazole, for fungal infections ^18^.

In light of the emerging utility of Hos2 inhibition as an anti-fungal strategy, we have cloned and characterized the *C. albicans HOS2*. In this report, we present details on optimizing the codons and cloning for heterologous gene expression in Sf9 insect cells. Purified Hos2 was characterized by functional deacetylase activities on histone/tubulin preparations and inhibition studies with SAHA, TSA and MS-275. The Candida genome database reports Hos2 to be a putative NAD+ dependent histone deacetylase ^23^, reminiscent of sirtuins. The Candida genome encodes for 3 sirtuins (HST1, HST2 and HST3). It has been shown that inhibition of HST3 by either gene repression or nicotinamide treatment reduces to a considerable extent the clinical severity of candidiasis ^24^. In light of this reported observation, we checked for sirtuin like activity of purified Hos2 preparations using resveratrol, which activates sirtuins ^25^.

## Materials and methods

### Isolation of *C. albicans* genomic DNA

*C. albicans* ATCC 90028 was obtained from ATCC and grown in Sabouraud dextrose media. The protoplasts were prepared from an overnight culture of fully-grown mycelia using zymolyase (Cat. No. L5263, Sigma, St. Louis, MO, USA) as per the manufacturer’s instructions. Protoplasts were lysed in a chaotrophic salt solution and genomic DNA was isolated according to manufacturer’s instruction (Qiagen, Hilden, Germany). The final eluate was reprecipitated with ammonium acetate and isopropanol and DNA quantified by UV spectrophotometer (Spectramax Gemini XS, Molecular devices, CA. USA).

### Cloning and expression of HOS2 in an insect cell expression system

Oligos were designed with codon changes made for 4 ^th^ and 271 ^st^ serine residues. Full length *HOS2* gene was amplified by using 4 different primers ( Table 1) using splicing by overlap extension (SOE PCR), so that codon usage could be maintained in any heterologous expression system. The full-length blunt end PCR product was cloned in to pJET1.2 cloning vector (CloneJET PCR Cloning Kit Thermo Scientific) and confirmed by restriction digestion using *BamH1* and *Not1*. DNA sequencing using T7 promoter (forward, 5´-TAATAC GACTCACTATAGGG-3´) and pJET1.2 (reverse, 5´-AAGAACATCGATTTTCCATGGCAG-´3) sequencing primer ascertained the sequence of the recombinant Hos2 gene.

**Table 1.** Oligonucleotide primer pairs for the PCR amplification of Hos2 gene by SOE PCR.

Sl. No.	Primer Name	Primer sequence
1	PR1	5´ AAAAGGATCCATGACGATA **TCC**ATAAGTGAAACAGATACG 3´
2	PR2	5´ ATTATTTAAA **TCC**ATTATGGAACCGTTAAT 3´
3	PR3	5´ ATTAACGGTTCCATAAT **GGA**TTTAAATAAT 3´
4	PR4	5´ CACCACTAGCGGCCGCTAAGTCATTAGTTCTCCTAGTTTGGTTTCA 3´

Recombinant baculoviruses were generated using the Bac-to-Bac ^®^ baculovirus expression system according to the instructions of the manufacturer (Cat. No. 10359-016, Invitrogen, Carlsbad, USA). Sf9 insect cells (Cat. No. B825-01, Invitrogen, Carlsbad, USA) were cultured in complete TMN-FH medium (Cat. No. 554760, BD Bioscience, NJ, USA) served as host cells for virus generation and/or protein production. The *HOS2* gene was cloned in frame with N-terminal hexa-histidine tag into the transfer vector pFastBac-HT B (Cat. No. 10584-027, Invitrogen, Carlsbad, USA) and transformed in to *Escherichia coli* DH10Bac cells (Cat. No. 10361-012, Invitrogen, Carlsbad, USA). Sf9 cells were transfected using cellfectin reagent (Cat. No. 10362-100, Invitrogen, Carlsbad, USA) with the recombinant bacmid, and the resulting viruses were tested for their ability to produce recombinant Hos2 protein using western blot. Production cultures were performed T-150 cell culture flasks (Greiner bio-one GmbH, Germany) at a density of 16×10 ^6^ cells per flask. The cultures were inoculated with recombinant baculovirus stocks at multiplicities of infection of 10. At 4 days after infection, cells were harvested by centrifugation at 1200 g for 15 min at 4°C in a tabletop centrifuge (Model 5804R, Eppendorf AG, Hamburg, Germany). Cell pellets were stored at -80°C until protein purification.

### Purification of recombinant Hos2 protein

The infected Sf9 cell pellets were resuspended in in-house ice-cold lysis buffer [10 mM Tris pH 7.5, 130 mM NaCl, 1% triton X-100, 10 mM NaF, 10 mM NaPi (sodium phosphate), and 10 mM NaPPi (sodium pyrophosphate)] with 1 X EDTA free protease inhibitor cocktail (Cat. No. 539134, Calbiochem, Merck-Millipore, San Diego, CA). Insoluble material was removed by centrifugation (Model 5804R, Eppendorf AG, Hamburg, Germany) at 16000 g, 4°C for 15 min. The cleared lysate was processed for nickel affinity chromatography. Briefly, the cell supernatant was loaded onto a 3-ml nickel-nitrilotriacetic acid-agarose resin (Ni-NTA agarose, Cat. No. 30210, Qiagen, Hilden, Germany) packed column pre-equilibrated with equilibration buffer (25 mM Tris-Cl pH 8.0, 300 mM NaCl). The column was washed with wash buffer (25 mM Tris-Cl pH 8.0, 300 mM NaCl, 20 mM imidazole). His-tagged Hos2 protein was eluted with buffer containing 300 mM imidazole (Sigma, St. Louis, MO, USA). The Hos2 protein preparation was dialyzed into buffer (25 mM Tris, pH 8.0, 138 mM NaCl, 10% glycerol) and kept in 100 μl aliquots at -70°C. The concentration of Hos2 protein in the final eluate was estimated by Bradford assay (Bio-Rad Laboratories, CA, USA).

### Hos2 deacetylase enzymatic assay

HDAC inhibitors, namely SAHA, TSA or MS-275 were dissolved in DMSO as 10 mM stock and subsequently diluted in 1X assay buffer (50 mM Tris Cl, pH 8.0, 137 mM NaCl, 2.7 mM KCl, 2.5 mM MgCl _2_, 1 mg/ml BSA). Enzymatic assay was carried out in triplicates using the fluorogenic Class I HDAC substrate, Boc-Lys (Ac)-AMC (Cat. No. I1875, Bachem AG, Bubendorf, Switzerland). Briefly, 0.5 μg of purified recombinant protein in a volume of 10 μl of assay buffer was incubated with 50 μl of appropriate concentration of HDAC inhibitors and 20 μM of substrate at 37°C for 1 hr in 100 μl reaction volume. Reactions were terminated by the addition of trichostatin A (TSA)/suberoylanilide hydroxamic acid (SAHA), trypsin (1 mg/mL) (Sigma, St. Louis, MO, USA) and left at 37°C for 15 min, before reading the plates in a fluorimeter (Spectramax Gemini XS, Molecular devices, CA. USA) at wavelengths 360 nm (ext) and 460 nm (emi).

### Production of polyclonal anti sera against Hos2 protein

Female BALB/c mice were purchased from The Jackson Laboratory (Bar Harbor, ME) at 4 weeks of age and then housed at the animal facility, Orchid Chemicals and Pharmaceuticals for 2 weeks in a specific-pathogen free facility with a 12 h light cycle (6 am–6 pm) and a 12 h dark cycle (6 pm–6 am). Groups of four mice were housed in sterilised polypropylene cages covered with stainless steel grid top, lined with autoclaved clean rice husk bedding. All animal experimentations were approved by the institutional animal ethics committee (Protocol No. 01/IAEC-05/PPK/2009). The native Hos2 protein (expressed in pET-32 bacterial vector system and purified using nickel affinity chromatography under denaturating conditions) was emulsified in complete Freund’s adjuvant and injected subcutaneously into two female BALB/c mice (20 μg/mice). Booster doses of the deacetylase antigen (20 μg/mice) were given on the 14 ^th^ and 21 ^st^ day in Freund’s incomplete adjuvant. Mice were bled 7 days after the second booster dose and polyclonal anti sera was separated after clotting the blood ^26^.

### Cell culture

Jurkat, a human T lymphocyte cell line, and HeLa, a human cervical adenocarcinoma cell line was obtained from ATCC and were cultured in DMEM (Gibco, Life technologies) supplemented with 10% (v/v) fetal calf serum (FCS), 2 mM glutamine, 100 units/ml penicillin and 100 µg/ml streptomycin (Gibco, Life technologies).

### Isolation of nuclear histones from mammalian cells

Acetylated histones were isolated from HeLa cells treated with the HDAC inhibitor SAHA as per published protocol ^27^. The histone pellet was then resuspended in ultra pure water, stored in 50 μl aliquots at -70°C and the protein concentration was determined using a BCA kit (Pierce).

### Isolation of histones from *Candida sp*.

*C. albicans* ATCC 90028 mycelia (~5 gm wet weight) were washed with water, centrifuged at 10,000 rpm for 10 minutes at 4°C and the mycelial pellet was resuspended in 50 ml of 0.1 mM Tris-HCl, pH 9.4, 10 mM DTT. The sample was incubated with shaking at 30°C for 15 min and pelleted. The pellet was washed with 50 ml of sorbitol/HEPES buffer (1.2 M sorbitol, 20 mM Hepes, pH 7.4) and left resuspended in the same buffer containing lyticase (1000 units) overnight at 30°C for spheroplasting ^28^. The sample was pelleted and proceeded to histone isolation using acid extraction as described previously ^29^. Acetone was added at 3:1 (vol/vol) to precipitate the histones, which were subsequently dissolved in 10 mM Tris, pH-8.0.

### Deacetylation of nuclear histones

Acetylated histones isolated from SAHA treated HeLa cells were used for the deacetylation assay with recombinant Hos2 enzyme. In brief, purified acetylated histones (2 μg) were incubated with different amounts of recombinant Hos2 enzyme. Histone deacetylation with 300 ng of rhHDAC1 or rhHDAC6 (expressed in-house using Baculo-viral expression system) was used as a positive control. Deacetylation assays were carried out in 100 μl reaction volume for 1 hr at 37°C in reaction buffer (50 mM Tris Cl, pH 8.0, 137 mM NaCl, 2.7 mM KCl, 2.5 mM MgCl _2_, 1 mg/ml BSA). At the end of incubation, the reaction was stopped by the addition of 1X Lammeli sample buffer. The protein samples were resolved by SDS-PAGE and immunoblotted with anti-acetylated H3 Histone (Ac-K-9) antibody to study the deacetylation of H3-histone by rHos2. The assay results are reproducible in three independent experiments.

### Deacetylation of acetylated tubulin

Whole cell extracts from TSA (Sigma, St. Louis, MO, USA) treated Jurkat cells were used for the α-tubulin deacetylation assay with recombinant Hos2 enzyme. In brief, whole cell extract (10 μg) were incubated with 5 and 8 μg of recombinant Hos2 protein. Deacetylation assays were carried out in 100 μl reaction volume for 3 hr at 37°C in reaction buffer (50 mM Tris-Cl, pH 8.0, 137 mM NaCl, 2.7 mM KCl, 2.5 mM MgCl _2_). At the end of incubation, the reaction was stopped by the addition of 1X Lammeli sample buffer. The protein samples were resolved by SDS-PAGE and immunoblotted with either anti-acetylated α-tubulin or anti-α-tubulin antibodies. The assay results are reproducible in two independent experiments.

### Western blotting

Recombinant Hos2 protein or the acetylated/deacetylated nuclear histones from deacetylation assays were separated on 10–15% SDS-polyacrylamide gels. The gels were stained with coomassie blue (Phast gel, Aersham) or electro-blotted onto nitrocellulose membrane (HyBond-C, Amersham), which was blocked with 5% nonfat dry milk (Cat No. 70166, Fluka, Sigma-Aldrich) in 0.1% Tween- 20 in TBS for 1 hr, followed by overnight incubation at 4°C with either the polyclonal mouse serum (dilution 1:4000 of the sera), rabbit polyclonal anti-acetylated H3-Histone (dilution 1:4000, Ac-K-9 of H3-Histone, Cat. No. 06-599; Millipore), mouse monoclonal anti-acetylated tubulin (dilution 1:15000, Cat. No. T7451, Sigma) or mouse monoclonal anti-tubulin antibody (dilution 1:150000, Cat. No. T6199, Sigma). After incubation with the appropriate horseradish peroxidase conjugated secondary antibodies (Bovine anti-mouse IgG HRP conjugate-dilution 1:7500, Santa Cruz, SC-2371, Goat anti-rabbit IgG-HRP Upstate (Millipore) 12–348, dilution 1:4000), supersignal west pico substrate (Thermo Scientific Pierce) was used for detection.

### *In vitro* Sirt1 activation assay

HeLa nuclear extract (2 μg) or recombinant Hos2 enzyme (300 ng) was incubated with NAD+ (500 μM), Fluor de Lys ^®^−Sirt1 (Enzo lifescience), varying concentrations of resveratrol (Cat. No. 0219605205, MP Biomedicals, Ohio, USA) (30, 100, 250 and 500 μM) in presence or absence of the pan-HDAC inhibitor trichostatin, in 50 μl reaction volume at 37°C for 30 min. The reaction was carried out in triplicate following manufacturer’s protocol (Enzo lifescience).

### Statistical analysis

All values are expressed as mean ± standard deviation and the graphs were generated using Graph-Pad Prism ^®^ (Version 4) for Windows (GraphPad Software, San Diego, California, USA. Statistical analysis was performed by one-way analysis of variance (ANOVA), followed by Bonferroni multiple comparison test for all parameters. Results were considered statistically significant at P < 0.05.

## Results

### HOS2 gene PCR

Codon usage in *C. albicans* is different from standard genetic code. The 4 ^th^ serine (CUG) and the 271 ^st^ serine (CUG) of *HOS2* are translated as leucine in both mammalian and in insect cell expression systems. Hence, these codons were mutated using oligonucleotide primers to express serine in the recombinant Hos2 protein. The PCR product was cloned in the pFastbac-HTB shuttle vector and subsequently into baculo viral DNA using the Bac-to-Bac expression system.

### Protein expression and purification

**Figure 1.**
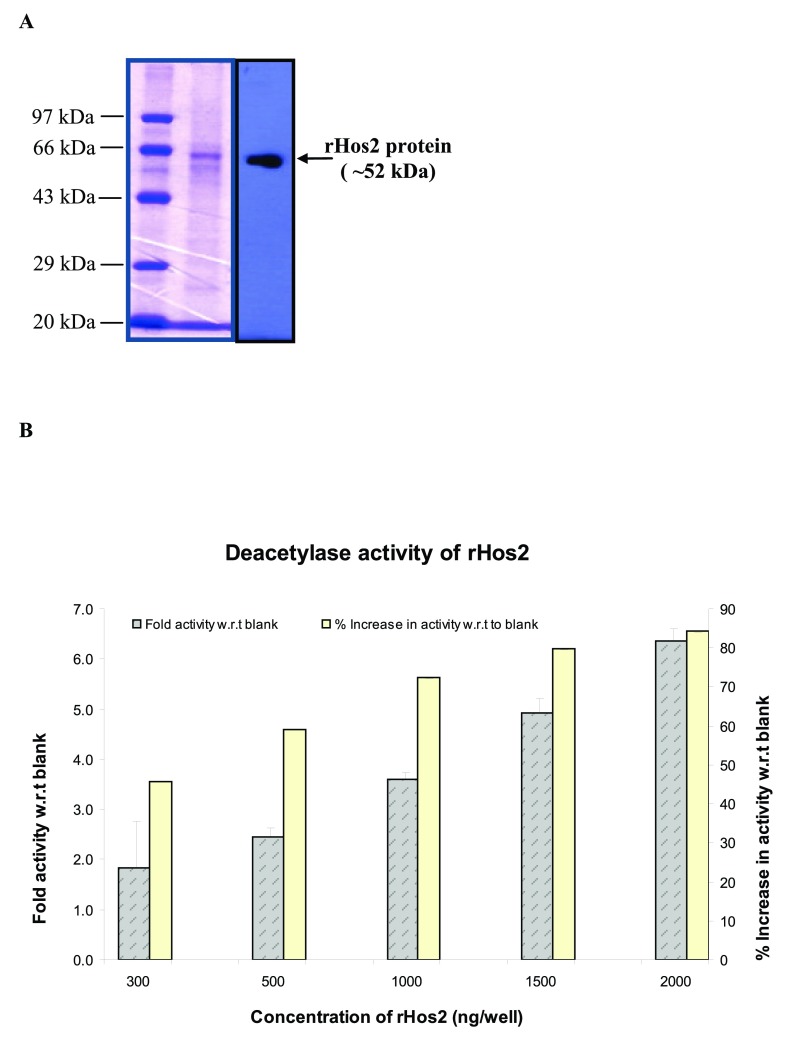
Expression of his-tagged Hos2 in insect cells and its characterization. ( **A**) Sf9 cells were infected with 10 MOI of recombinant Hos2 baculovirus for 72 hours and the soluble fraction was collected. Two μg of protein was separated on a 10% SDS-PAGE. Lane 2 is coomassie stained SDS-PAGE and Lane 3 is Hos2 protein recognized by polyclonal anti sera from mice. Molecular weight markers are labeled at the left side (Lane 1) of the blot. ( **B**) Concentration dependent increase in deacetylase activity following incubation of baculo expressed Hos2 with Boc-lys (ac)-AMC fluorogenic peptide substrate in 1 hr assay at 37°C. Assays were carried out in triplicates and analysed for statistical significance by one way ANOVA (Bonferroni’s Multiple Comparison Test) using GraphPad Prism software.

The Hos2 enzyme was expressed in the baculoviral-insect cell expression system as a NH _2_-terminal hexa histidine tagged fusion protein, which is detected on Western blot as a ~52 kDa protein using our own polyclonal anti-Hos2 anti-sera raised against Hos2 protein in mice. SDS-PAGE analysis of purified protein revealed a major band at ~52 kDa ( Figure 1A).

Tubulin deacetylation and rHos2 protein blots Western blot analysis of rHos2 protein polyclonal anti-sera: Western blot analysis of rHos2 protein using polyclonal antisera raised from mice.
Tubulin Deacetylation Blot data_F1000: Western blot analysis for tubulin Deacetyation by rHos2 protein.

### *In vitro* deacetylation assay using synthetic peptide substrate

Recombinant Hos2 enzyme was assayed for deacetylase activity using the synthetic deacetylase substrate, Boc-Lys (ac)-AMC. The total activity with Boc-Lys (ac) AMC showed the enzyme to be active in deacetylating the lysine residue and the activity increased significantly ( *P < 0.05*) with an increase in Hos2 concentration ( Figure 1B).

**Figure 2.**
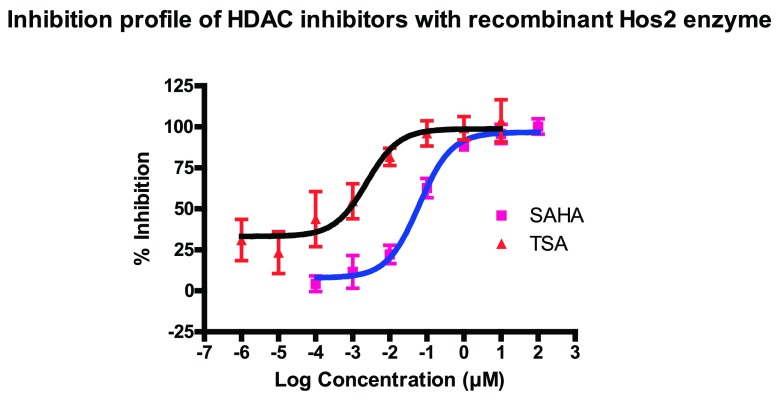
Dose-dependent inhibition of Hos2 deacetylase activity by SAHA (squares) and TSA (triangles). Recombinant Hos2 enzyme was incubated with different concentrations of pan-HDAC inhibitors (SAHA or TSA) and the enzyme activity assay was performed. Assays were carried out in triplicates and error bars were calculated using GraphPad prism software.

The inhibition of deacetylation activity of recombinant Hos2 was studied using classical HDAC inhibitors namely SAHA, TSA and MS-275. TSA was very potent in inhibiting Hos2 with an IC _50_ of 2.8 nM, SAHA inhibited Hos2 with an IC _50_ of 65 nM ( Figure 2, Table 2). However, MS-275 showed > 50% inhibition of Hos2 activity only at 10 μM ( Table 3).

**Table 2.** IC _50_ values of standard HDAC inhibitors.

Sl. No.	Pan HDAC inhibitor	IC _50_ ± SE (nM)
1	SAHA	65.4 ± 2.4
2	TSA	2.8 ± 0.9

**Table 3.** Hos2 enzyme inhibition values of Class I HDAC inhibitor.

Sl. No.	Class I HDAC inhibitor	% inhibition ± SE	
		10 µM	1 µM
1	MS275	56.3 ± 0.9	46.3 ± 1.4

### *In vitro* deacetylation assay using natural substrates

**Figure 3.**
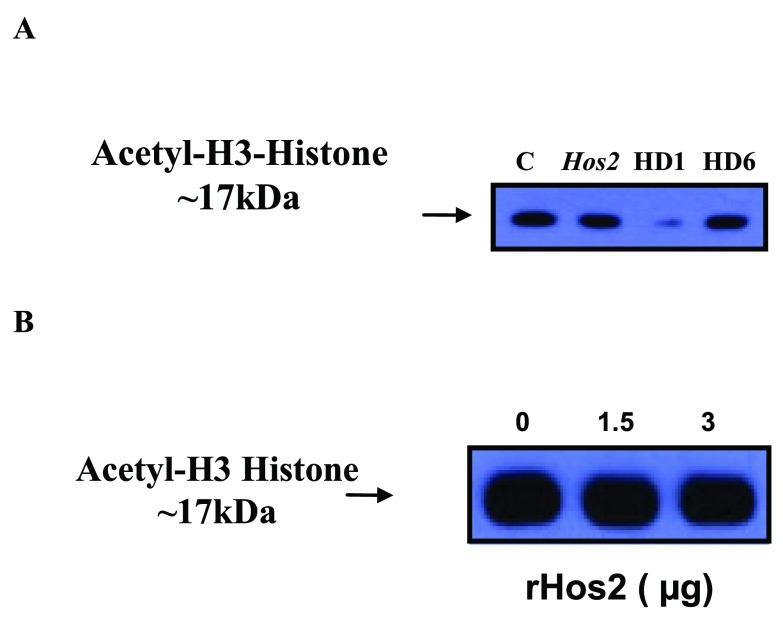
Deacetylation assays of deacetylases with nuclear histones. **A**. Histone H3 acetylation determined by Western blot of extracted acetylated histones (2 µg) Control (Lane 1), incubated with 300 ng of recombinant Hos2 protein (Lane 2), recombinant human HDAC1 (HD1 Lane 3) and recombinant human HDAC6 (HD6 Lane 4) for 1 hour at 37°C in HDAC assay buffer. **B**. Histone H3 acetylation determined by Western blot of extracted acetylated histones (2 µg) isolated from *C. albicans*, incubated with different concentrations of recombinant Hos2 protein for 1 hour at 37°C in HDAC assay buffer.

**Figure 4.**
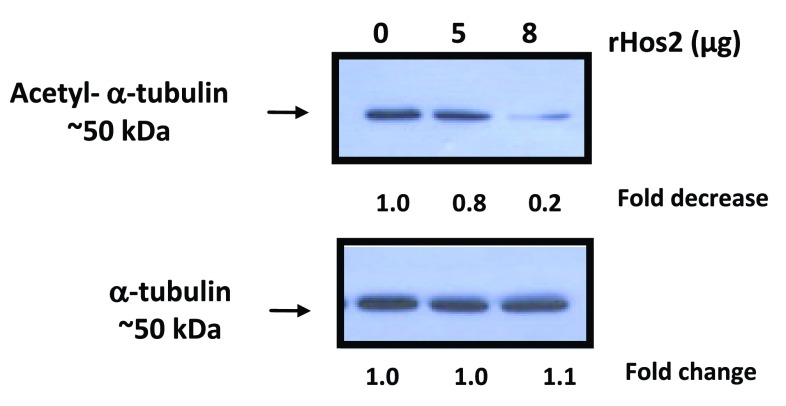
Deacetylation assays of rHos2 with α tubulin. α-tubulin acetylation determined by Western blot of SAHA-treated whole cell extracts containing acetylated α-tubulin (10 µg) incubated with 0, 5 or 8 µg of recombinant Hos2 for 3 hrs at 37°C in HDAC assay buffer.

The ability of purified Hos2 protein to deacetylate acetylated histones was examined *in vitro* using acetylated nuclear histone preparation made from SAHA treated HeLa cells. The nuclear histones from HeLa cells were isolated using a modified protocol of Shechter *et al.* ^27^ and established the deacetylation assay using rhHDAC1/rhHDAC6 as controls along with rHos2. In these assays it was found that 0.3 μg of rhHDAC1 was able to deacetylate the nuclear acetylated histones as detected by an anti-H3-K9 histone antibody. Recombinant hHDAC1 was more potent in deacetylating lysine residues in H3-histones than rhHDAC6. However, no significant deacetylation of H3-Histone was seen with 0.3 μg of Hos2 ( Figure 3A). Similar results were obtained with acetylated histones from *Candida* in the *in vitro* histone deacetylation assay with purified Hos2 protein (up to 3 μg, Figure 3B). In contrast, when recombinant Hos2 was incubated with mammalian acetylated α-tubulin, a significant reduction in acetylation of α-tubulin (K40) could be observed ( Figure 4).

Deacetylase activities of rHos2 and dose response curves of rHos2 inhibition by standard HDAC inhibitors: UPDATE 1 Dose response curve of rHos2 enzyme inhibition by MS275: Fluorimetric deacetylase assay with rHos2 protein using Boc-Lys(ac)-AMC substrate in presence of different concentrations of MS-275.
Dose response curve of rHos2 enzyme inhibition by SAHA: Fluorimetric deacetylase assay with rHos2 protein using Boc-Lys(ac)-AMC substrate in presence of different concentrations of suberoylanilide hydroxamic acid (SAHA).
Dose response curve of rHos2 enzyme inhibition by TSA: Fluorimetric deacetylase assay with rHos2 protein using Boc-Lys(ac)-AMC substrate in presence of different concentrations of trichostatin.
rHos2 conc_Enzyme activity: Fluorimetric deacetylase assay with rHos2 protein using Boc-Lys(ac)-AMC substrate. Updated from: http://dx.doi.org/10.6084/m9.figshare.841655

### *In vitro* Sirt1 deacetylation assay using fluor-de-lys substrate

**Figure 5.**
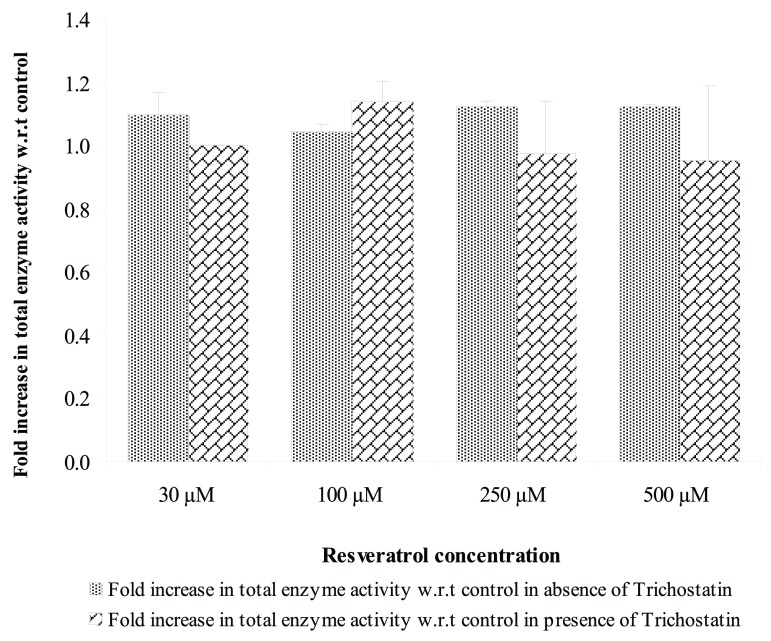
SIRTI activation following incubation of Resveratrol with rHos2. Fold activation of the Sirt1 like activity following incubation of recombinant *Hos2* enzyme with different concentration of resveratrol in presence and absence of HDAC inhibitor trichostatin. Assays were carried out in triplicates and analysed for statistical significance by one way ANOVA (Bonferroni’s Multiple Comparison Test) using GraphPad Prism software.

Since Hos2 is a putative NAD+ dependent deacetylase, a feature of sirtuin class of deacetylases, it was of interest to check if the Hos2 protein displayed any sirtuin like activity. The sirtuin-like activity in response to the sirtuin activator resveratrol was studied using fluor-de-lys, a synthetic Sirt1 substrate. HeLa nuclear extract was used as a positive control for sirtuin activity. In the presence of TSA where the HDAC activities are inhibited no appreciable NAD+ dependent deacetylase-like activity was seen, following incubation of Hos2 with different concentrations of resveratrol ( Figure 5).

Sirtuin assay of purified Hos2 preparations using resveratrol: UPDATE 1 Fluorimetric Sirtuin1 like deacetylase assay with rHos2 protein using Fluor de Lys®−Sirt1 substrate in presence of Sirtuin1 activator Reserveratrol. This experiment is carried out in presence of HDAC inhibitor Trichostatin to inhibit any residual Histone deacetylase activity. Updated from: http://dx.doi.org/10.6084/m9.figshare.841658.

## Discussion

Pathogenic fungi are increasingly responsible for life threatening infections in the elderly and immunocompromised patients. While some species have intrinsic resistance to anti-fungals, others develop resistance during the course of treatment. Increasing antifungal resistance and treatment failures in patients is becoming a challenge.

The *Candida* genome encodes at least 3 distinct classes of histone deacetylases in addition to sirtuins. There are 8 different histone deacetylases ( *HOS1, HOS2, HOS3, HDA1, HDA2, HDA3, RPD3, RPD31*) which all have distinct roles in the morphogenesis of *C. albicans*. HDAC inhibitors, by virtue of their ability to prevent antifungal resistance *in vitro,* have been proposed as antifungal adjuvants. Hos2 is a histone deacetylase and interacts with several proteins both in the cytoplasmic milieu as well in the nucleus ^30^. Hos2 along with Set3 (SET domain-containing protein 3) an associated protein, plays an important role in controlling gene expression by associating with transcriptionally active regions of the chromatin ^31^.

It has been surmised that inhibiting Hda1 for example might enhance the anti-fungal effect of HDAC inhibitors by limiting hyphal development, while inhibiting Hos2 might contribute to limiting yeast development ^32^. Hos2 has an essential function in morphogenesis especially during conditions of nitrogen starvation ^33^. The critical role of HDAC`s in *C. albicans* pathogenesis and survival to antifungal treatment underscores the necessity to study HDAC function in this organism.

The increasing clinical incidences of azole resistant fungal infections in critical care patients, makes a good reason to find additional drug targets to control such diseases. There is at least one small molecule (MGCD 290) that inhibits Hos2 histone deacetylase that has progressed to clinical trials. MGCD 290 in combination with azoles was shown to be active against azole resistant yeasts and moulds ^18^.

In order to better understand the role played by the *Candida* Hos2 enzyme we attempted to clone, express and characterize the protein in detail. This study describes the cloning, expression, purification and characterization of the Hos2 deacetylase enzyme from *C. albicans*.

We cloned and expressed the *HOS2* gene in baculoviral expression system as a 6x his-tagged protein, which exhibits classical deacetylase activity with the synthetic Boc-Lys (ac)-AMC peptide substrate. In our study, the yield of the Hos2 protein was generally low and probably could be attributed to difference in codon usage between *Candida* and Sf9 insect cells. This *in vitro* enzymatic assay, amenable to high throughput, could be used for screening potent fungal Hos2 inhibitors that could be a potential anti-fungal adjuvant.

Our studies with the recombinant Hos2 protein showed that it is susceptible to inhibition by standard HDAC inhibitors such as SAHA and TSA. We characterized the inhibition profile of purified proteins with SAHA and TSA and showed that TSA is a more potent inhibitor of Hos2 with an IC _50_ of 2.8 ± 0.9 nM compared to SAHA (IC _50_ 65.4 ± 2.4 nM). Our studies with the Class I HDAC inhibitor MS-275 showed that this inhibitor did not inhibit Hos2 deacetylase as effectively as the pan HDAC inhibitors SAHA or TSA, suggesting that *Candida* Hos2 is more similar to Class II deacetylases.

The recombinant Hos2 failed to deacetylate either mammalian or fungal nuclear histones, suggesting that the histones are not the preferred substrates for the Hos2 enzyme. The fact that, the recombinant Hos2 enzyme did not show any inhibition with the Class I inhibitor MS-275 led us to explore alternate substrates including tubulins, which are substrates for Class II histone deacetylases. Experiments with total lysates from Jurkat cells containing acetylated α-tubulin showed a dose dependent deacetylation albeit at higher concentration of Hos2 (> 5 μg). Hos2 in essence resembles the Class II mammalian HDACs, specifically HDAC6 in its preference for tubulin deacetylation. It has been shown that microtubules in the fungal hyphae drive nuclear dynamics and cell cycle progression to morphogenesis ^34^. In view of the fact that Hos2 seems to preferentially deacetylate tubulins, it would be interesting to see if Hos2 inhibitors would act as anti-fungals, either as a monotherapy or in synergy, with existing anti-tubulin agents such as benomyl, nocodazole etc. The physiological relevance of tubulin deacetylation by Hos2 warrants further study.

The *Candida* genome database ^23^ predicts Hos2 protein to be a NAD+ dependent deacetylase. Sirtuins which are classified as Class III deacetylases are NAD+ dependent enzymes activated by polyphenols such as resveratrol. Sirtuins have been proposed to regulate cellular metabolism, ageing and other related processes, specifically cellular stress response to caloric restriction, mediating life span extension. The role of resveratrol as a sirtuin activator has been resolved recently and it is now known that in addition to activating Sirt1, it also activates Sirt5, while inhibiting Sirt3 ^25^. Thus inhibiting any sirtuin like activity with small molecule inhibitors could be another way of enhancing the activity of currently used anti-fungals. We evaluated the possibility of Hos2 being a sirtuin like enzyme with a known Sirt1 activator resveratrol. We did not observe any significant ( *P* value 0.5317 and 0.4411, in the presence and absence of trichostatin respectively) activation of NAD+ dependent deacetylase activity with the fluor-de-lys substrate.

In conclusion this study establishes a functional assay for purified Hos2 protein. This *in vitro* enzymatic assay can be used to screen small molecule inhibitors of Hos2, which can synergise current anti-fungals in the clinic.

## Data availability

figshare: Tubulin deacetylation and rHos2 protein blots, doi: 10.6084/m9.figshare.841666 ^35^

figshare: Sirtuin assay of purified Hos2 preparations using resveratrol: UPDATE 1, doi: 10.6084/m9.figshare.1031579 ^36^

figshare: Deacetylase activities of rHos2 and dose response curves of rHos2 inhibition by standard HDAC inhibitors: UPDATE 1, doi: 10.6084/m9.figshare.1031581 ^37^
