# Supplementary material for: Functional characterization of Candida albicans Hos2 histone deacetylase
Source: F1000Res. 2014 Jul 22;2:238. Originally published 2013 Nov 11. [Version 3] doi: 10.12688/f1000research.2-238.v3 (PMC4111124; doi:10.12688/f1000research.2-238.v3)
Supplement: Peer review history

# Peer review: Referee response for version 2

**DOI:** 10.5256/f1000research.4538.r5126

**Published:** 2014-06-26

**Authors:** Malcolm Whiteway

**Affiliations:** 1 Department of Biology, Concordia University, Montréal, QC, Canada

- Figure 1a protein purification is OK, and the the signal with the antibody to the purified protein is fine, but needs the control lane with extract from uninfected cells.
- The presentation of the activity in Figure 1b is a bit unorthodox.  Why not just present the blank and the experiment for each concentration, and if you want to present the fold activity with respect to blank, it can be a figure below the blank and experimental bars.
- Deacetylation assays support activity only with alpha tubulin, not histones.
- For the sirtuin assay, a positive control would be informative; can you see activation of a pure sirtuin in the assay performed.

Minor Comments - in the first line of the Results, it would be better to say the first CUG and 271 ^st^ CUG codon rather than serine.

I have read this submission. I believe that I have an appropriate level of expertise to confirm that it is of an acceptable scientific standard, however I have significant reservations, as outlined above.

# Response

**Published:** 2014-07-07

**Authors:** Karthikeyan Ganesan

**Affiliations:** Biology, Orchid Research Laboratories Ltd, India

The points raised by the referee are addressed in the new version of the article.

We have carried out a separate experiment to show that the signal in the western blot is specific (this can be downloaded here but is not included in the revised article).

Fig 1B was specifically modified at the request of the editorial team during the initial stage and subsequently modified based on inputs from other referee(s).

The title has now been modified to reflect that tubulins are deacetylated and not histones.

We used HeLa nuclear extracts as positive control for Sirtuin assay and Resveratrol did show Sirtuin activation.

Minor Comment regarding the mention of 1st and 271st CUG codon will be modified in the revised article.

# Peer review: Referee response for version 2

**DOI:** 10.5256/f1000research.4538.r4908

**Published:** 2014-06-11

**Authors:** Donna MacCallum

**Affiliations:** 1 Aberdeen Fungal Group, University of Aberdeen, Aberdeen, UK

Authors have addressed prior concerns.

I have read this submission. I believe that I have an appropriate level of expertise to confirm that it is of an acceptable scientific standard.

# Peer review: Referee response for version 1

**DOI:** 10.5256/f1000research.2168.r4017

**Published:** 2014-04-16

**Authors:** David Soll

**Affiliations:** 1 Department of Biology, University of Iowa, Iowa City, IA, USA

This manuscript describes an *in vitro* assay strategy for testing the effects of antifungal compounds on the activity of Hos2 deacetylase large scale screens.The goal is to find compounds that could be used as facilitators in combinatorial drug treatments for azole resistant *Candida albicans* infections. Although *in vitro* assays are sometimes useful for developing *in vivo* strategies, they are not ideal for screening and identifying optimal drugs.

As shown in this study by the authors, the recombinant Hos2 they generated exhibits paradoxically tubulin-specific deacetylase activity and not Hos2 specific activity. This finding seems to undermine their intent, because anti-tubulin deacetylase activity or anti-human HDAC6 activity demonstrated *in vitro* could be an artifact of the recombinant protein. In addition, anti-tubulin and anti-HDAC6 activity might result in adverse side effects on human host functions and thus affect the specificity of the primary drug.

It is surprising that the recombinant Hos2 did not show any activity in acetylating either the human or *Candida* histone H3 *in vitro*, despite the fact that several earlier studies in *C. albicans *has demonstrated that Hos2 is involved in morphogenetic programs by affecting chromatin function. In addition, an isolated study has demonstrated the efficacy of a Hos2-specfic inhibitor on azole-resistant strains of *C. albicans* (Pfaller *et al.*, 2009), which is quoted by the authors. The authors do state in the introduction that Hos2 is not a typical deacetylase and has been described by the *Candida* Genome Database as a member of NAD-dependent sirtuins. The authors’ data show that their recombinant version lacks this activity, but does exhibit inhibition by classical inhibitors of histone deacetylases.

In addition to this major problem there are a few other aspects of the paper that need to be addressed:

- Firstly, Figure 1 and Figure 5 do not have error bars to indicate reproducibility of the data. Also, a statistical analysis of data sets would be useful to indicate the significance of the results.
- Secondly, the units in Figure 2 and Table 2 need to be consistent with the units described in the text.
- Thirdly, since Figure 4 shows the effect of recombinant Hos2 on only tubulin, the title needs to be changed to indicate that the assay was not done with purified histones.
- Finally, it is not clear what *“fold activity”* (in Figure 1B) and *“fold activation” *(Figure 5) refer to. 

I have read this submission. I believe that I have an appropriate level of expertise to confirm that it is of an acceptable scientific standard, however I have significant reservations, as outlined above.

# Response

**Published:** 2014-05-13

**Authors:** Karthikeyan Ganesan

**Affiliations:** Biology, Orchid Research Laboratories Ltd, India

As a pharmaceutical organization, involved in drug discovery, our initial compound libraries are screened for *in vitro* activity/inhibition assays. These *in vitro* assays act as important filters removing in-active compounds to progress only the most potent ones to *in vivo* screening.

Deacetylase assays using recombinant Hos2 enzyme and synthetic substrate Boc (Lys) AMC showed that the enzyme is active in standard fluorogenic assays.  Absence of deacetylase activity with histone preparations (Human/ *Candida*) in the *in vitro* histone deacetylation assays was a surprise but the fact that Class I HDAC inhibitor like MS-275 did not inhibit rHos2 in standard fluorogenic assays, led us to explore alternate substrate, namely tubulins.

We do agree with the reviewer that anti-tubulin deacetylase activity could be an artifact of recombinant protein, but we strongly feel that it is a remote possibility.

Any small molecule that shows anti-tubulin or anti-HDAC6 activity would be picked up in the *in vitro* screen and would be prevented from moving further up the discovery path. (Which can be re-purposed for different therapeutic )

We are indeed aware of the previous studies showing the effect of Set3/Hos2 complex and the opposing roles of Hda1 and Hos2 in *Candida *morphogenesis as well as the role of Hos2 in S. cerevisiae. In these studies Histone H4 acetylation levels vary dramatically, especially H4-K16, however the Histone H3 levels more or less remain constant, (supplementary materials from Wang *et al.*, 2002) and that`s probably the reason why we don`t see any dramatic deacetylation when probed with Histone H3-K9. The study by Pfaller *et al.* did have *in vivo* data, but did not have any *in vitro* data.

Error bars and statistical analysis have been incorporated for Figs. 1 and 5. Units for Fig. 2 and Table 2 corrected.

Label has been changed for Fig. 4

Fold activity and Fold activation is with respect to Blank and has been reflected in the figures.

# Peer review: Referee response for version 1

**DOI:** 10.5256/f1000research.2168.r4016

**Published:** 2014-03-19

**Authors:** Donna MacCallum

**Affiliations:** 1 Aberdeen Fungal Group, University of Aberdeen, Aberdeen, UK

This is an interesting study designed to examine the biological activities of *Candida albicans *Hos2 enzyme, a putative histone deacetylase. The authors express a recombinant version of the protein, which has a His tag added to the N-terminus, and use this to explore potential substrates for the enzyme.

There are a number of major issues with the data as presented, which need to be addressed:

- It is difficult to make any conclusions from the quantitative data presented in the figures as there are no error bars and the number of replicates is not stated. In addition, it is unclear what the data in Figures 1B and 5 has been expressed relative to (i.e. fold change relative to...?)  Statistical analyses should also be carried out to determine whether any differences are statistically significant ones.
- The authors should also revisit the data presented in Figure 2 and Table 2. The data does not correlate, as the authors suggest that the IC _50_ values are in micromolar amounts, yet the data in the figure would suggest millimolar levels - units should be checked.

- The title for Figure 4 should be modified, since this figure contains only data for beta-tubulin. 

- In the main text the authors need to be much clearer regarding the rationale for looking at tubulin as a substrate. The authors may also wish to consider the possibility that the substrate specificity and/or activity may have been affected by the N-terminal tag and how this could be investigated. 

- The discussion section should be revisited to remove the reiteration of the introduction and results. Instead, use this part of the paper to discuss the findings in relation to other published work. 

**Minor points:**

- *Candida albicans *is capable of causing superficial (oral and vaginal thrush) infections in immune normal hosts (abstract and introduction).

- Gene names, e.g. *CDR1 *and *ERG11*, should be in italics.

- For the enzymatic assay it would be good to state the volume of the assay so that final concentrations can be worked out.

- Figure and table legends should include the number of replicates carried out to generate the data.

I have read this submission. I believe that I have an appropriate level of expertise to confirm that it is of an acceptable scientific standard, however I have significant reservations, as outlined above.

# Response

**Published:** 2014-05-13

**Authors:** Karthikeyan Ganesan

**Affiliations:** Biology, Orchid Research Laboratories Ltd, India

Error bars have been incorporated and where possible, statistical analysis done and reported.

Error in reporting IC _50_ value in Table 2 and Fig. 2 is corrected.

Title for Fig. 4 changed to reflect tubulin deacetylation.

The rationale for choosing tubulin as a substrate has been explained. We agree with the reviewer that N-terminal tag could perhaps play a role, although we consider that as a remote possibility.

Minor points suggested by reviewer has also been addressed.
